# Supplementary material for: Cryo-EM Structures of Chronic Traumatic Encephalopathy Tau Filaments with PET Ligand Flortaucipir
Source: J Mol Biol. Author manuscript; Available in PMC 2023 Dec 1. (PMC7615338; doi:10.1016/j.jmb.2023.168025)
Supplement: Supplementary Data [file EMS191517-supplement-Supplementary_Data.pdf]

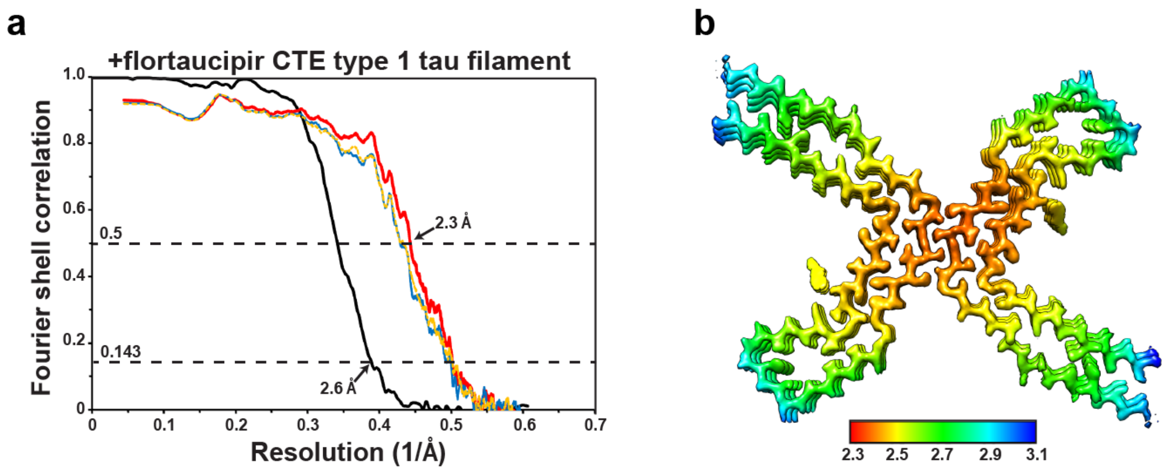


**Supplementary figure 1. Cryo-EM and model resolution assessment.**

a. Fourier shell correlation (FSC) curves for cryo-EM maps of the CTE Type I filaments with flortaucipir (in black); for the final refined atomic model against the final cryo-EM map (in red); for the atomic model refined in the first half map against that half map (in blue); and for the refined atomic model in the first half map against the other half map (in yellow). B. Top view of the cryo-EM density coloured by local resolution (in Å).

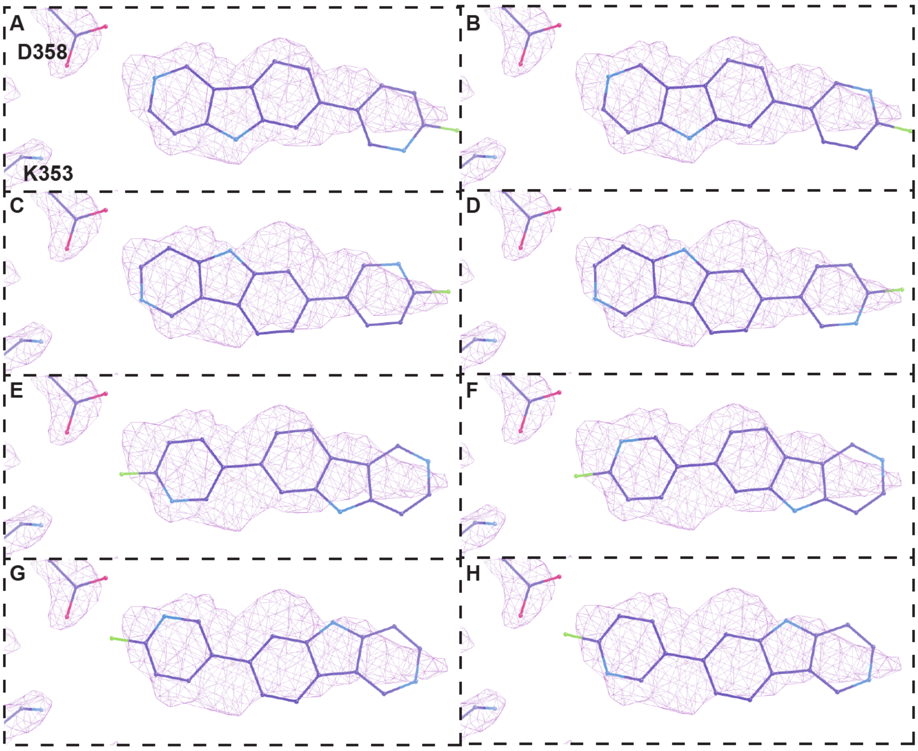


**Supplementary figure 2. Alternative dockings of flortaucipir.**

Eight alternatives for the docking of flortaucipir into the additional cryo-EM density are shown in panels A-H. The cryo-EM density alone cannot resolve which is the correct one. The conformation shown in panel A results in the best fit to the density. This conformation is also shown in Figure 2, and is the one used in PDB entry 8BYN.

**Supplementary Table 1**
**Cryo-EM data collection, refinement and validation statistics**

| | AD (+flortaucipir) | AD (-flortaucipir) | PART (+flortaucipir) | PART (-flortaucipir) |
|---|---|---|---|---|
| **Data collection and processing** | | | | |
| EM type | Titan Krios | Titan Krios | Titan Krios | Titan Krios |
| Magnification | 105,000 | 96,000 | 96,000 | 96,000 |
| Detector | K2 | K3 | Falcon 4 | Falcon 4 |
| Voltage (kV) | 300 | 300 | 300 | 300 |
| Electron exposure (e–/Å$^2$) | 60 | 51 | 40 | 40 |
| Defocus range (μm) | -1.0 to -2.5 | -1.8 to -3.0 | -1.0 to -2.0 | -1.0 to -2.0 |
| Pixel size (Å) | 1.15 | 0.828 | 0.824 | 0.824 |
| Symmetry imposed | C1 | C1 | C1 | C1 |
| Initial particle images (no.) | 673,528 | 656,157 | 385,082 | 1,123,147 |
| Final particle images (no.) | 114,174 | 561,329 | PHF:4,792<br>SF:42,917<br>CTE1: 58,019 | PHF:2,207<br>SF:36,686<br>CTE1: 48,163 |
| Map resolution (Å)<br>FSC threshold(0.143) | 2.70 | 2.68 | PHF:3.43<br>SF: 2.62<br>CTE1: 2.60 | PHF:3.66<br>SF: 2.70<br>CTE1: 2.69 |
| Helical twist (°) | 179.43 | 179.43 | PHF:179.46<br>SF: -1.08<br>CTE1: 179.43 | PHF:179.47<br>SF: -1.08<br>CTE1: 179.42 |
| Helical rise (Å) | 2.37 | 2.47 | PHF:2.37<br>SF: 4.77<br>CTE1: 2.37 | PHF:2.38<br>SF: 4.77<br>CTE1: 2.37 |
| **Refinement** | | | | |
| Initial model used (PDB code) | | | 6NWP | |
| Model resolution (Å)<br>FSC threshold (0.5) | | | 2.3 | |
| Map sharpening *B* factor (Å$^2$) | | | -70 | |
| Model composition | | | | |
| Non-hydrogen atoms | | | 3564 | |
| Protein residues | | | 450 | |
| Ligands | | | 6 | |
| *B* factors (Å$^2$) | | | | |
| Protein | | | 20.23 | |
| Ligand | | | 12.17 | |
| R.m.s. deviations | | | | |
| Bond lengths (Å) | | | 0.006 | |
| Bond angles (°) | | | 0.680 | |
| Validation | | | | |
| MolProbity score | | | 1.35 | |
| Clashscore | | | 6.29 | |
| Poor rotamers (%) | | | 0.00 | |
| Ramachandran plot | | | | |
| Favored (%) | | | 98.63 | |
| Allowed (%) | | | 1.37 | |
| Disallowed (%) | | | 0.00 | |

**Supplementary Table 2**
**Angles between the aromatic rings of residues in amyloid filaments and the helical axis**

| | | | | | | | | | |
|---|---|---|---|---|---|---|---|---|---|
| | **Tau** | | | | | | | | |
| | His299 | Tyr310 | His329 | His330 | Phe346 | His362 | His374 | Phe378 | |
| AD-PHF 7NRQ | | 44.176 | 45.859 | 40.835 | 44.121 | 39.190 | 54.349 | 47.266 | |
| AD-SF 7NRS | | 44.582 | 51.962 | 37.951 | 43.194 | 28.240 | 44.572 | 45.064 | |
| CTE-I 6NWP | | 47.375 | 40.383 | 26.322 | 42.627 | 19.847 | 40.393 | 40.486 | |
| PSP 7P65 | 35.573 | 42.101 | 37.285 | 53.369 | 47.948 | 28.432 | 46.576 | 45.556 | |
| | **Amyloid-β** | | | | | | | | |
| | Tyr10 | His13 | His14 | Phe19 | Phe20 | | | | |
| Aβ42-I 7Q4B | 32.177 | 42.470 | 47.405 | 44.436 | 41.556 | | | | |
| | **TDP-43** | | | | | | | | |
| | Phe283 | Phe289 | Phe313 | Phe316 | Trp334 | | | | |
| 7PY2 | 42.279 | 35.182 | 37.107 | 42.421 | 40.644 | | | | |
| | **TMEM106B** | | | | | | | | |
| Fold-I 7QVC | Tyr125 | Tyr132 | Tyr135 | Tyr143 | Tyr157 | Tyr158 | Phe171 | Tyr197 | Tyr209 |
| | 40.739 | 50.466 | 40.619 | 34.447 | 39.996 | 38.733 | 39.169 | 43.304 | 45.053 |
| | Tyr211 | Phe213 | His222 | Tyr236 | Phe237 | His239 | Tyr248 | Tyr250 | |
| | 39.602 | 32.289 | 39.651 | 38.169 | 33.657 | 26.803 | 37.038 | 40.669 | |
| Fold-III 7QWM | Tyr125 | Tyr132 | Tyr135 | Tyr143 | Tyr157 | Tyr158 | Phe171 | Tyr197 | Tyr209 |
| | 54.199 | 45.226 | 41.232 | 38.059 | 38.684 | 40.991 | 43.892 | 46.886 | 34.023 |
| | Tyr211 | Phe213 | His222 | Tyr236 | Phe237 | His239 | Tyr248 | Tyr250 | |
| | 35.416 | 32.141 | 36.487 | 39.556 | 35.345 | 22.204 | 42.375 | 41.373 | |
